# Revisiting the Newest Vital Sign Survey: Addressing Concerns About This Health Literacy Assessment Tool

**DOI:** 10.3928/24748307-20240515-02

**Published:** 2024-04

**Authors:** Jordy Schol, Luca Ambrosio, Yoshiyuki Yamada, Daisuke Sakai

In this Perspective, we shed light on the widely adopted Newest Vital Sign (NVS) assessment, a pivotal and commonly applied tool in evaluating health literacy. Despite its widespread adoption, we identified some critical issues that raise potential concerns surrounding its accuracy and applicability. The NVS survey was initially introduced by Weiss et al. (2005) as part of their innovative patient-reported measures called the “Newest Vital Sign.” This initiative was funded by the Pfizer pharmaceutical company for the Pfizer Clear Health Communication Initiative and has been promoted as one of their “most important contributions to the health literacy movement” (Pfizer, 2024). This screening assay is designed to evaluate patients' literacy specifically concerning health-related topics. The NVS involves participants reading and comprehending a fictitious ice cream nutrition label, followed by six related questions. Based on the number of correct answers, the survey provides a score reflecting the individual's health literacy, with a maximum score of 6, indicating higher health literacy levels. Overall, the study provides a simple and effective way for health care providers and researchers to assess patients' or general participants' health literacy and tailor their communication accordingly. The assay has been widely adapted (Smith et al., 2009; van der Vaart & Drossaert, 2017), including translations into various languages (Fransen, 2011), and is relatively easy to use. However, we have some concerns with the current format of the survey, particularly regarding the final two questions.

Question 5 asks if a patient with an allergy to peanuts could eat the fictitious ice cream, which lists peanut oil as an ingredient. According to the survey designers, the stated correct answer is “no.” Question 6 then asks why the participant answered “no” to question 5, with the correct answer being the “listing of peanut oil as one of the ingredients.” Answering “no” to question 5 and “peanut oil” to question 6 would result in the participant gaining 2 points toward their health literacy score, which constitutes 33% of the total and final score.

We would like to raise the possibility that the responses given to the food label presented in the survey may not be the correct interpretations. Peanut allergies are generally caused by the allergen Ara h1, which is found in the cupin-superfamily of proteins (Mueller et al., 2014). National health agencies, such as the U.S. Food and Drug Administration (FDA) and the European Food Safety Authority, require specific identification on food labels for products that may contain (traces of) peanuts to ensure the safety of individuals with allergies. However, this identification was not present on the fictitious label presented in the survey by Weiss et al. (2005). Furthermore, peanut oil is generally considered safe for individuals with a peanut allergy to consume, depending on the type of oil used. Refined peanut oil is a product that has had almost all traces of the peanut protein removed, and the risk of triggering an allergic reaction is considered extremely low (Blom et al., 2017; FDA, 2004). As such, the FDA does not require refined peanut oil to be labeled as an allergen on their food products (FDA, 2004). Unrefined peanut oils can still contain traces of peanut proteins, but large studies have found limited events of severe allergic responses caused by unrefined peanut oils, though consumption avoidance is recommended (Hourihane et al., 1997). Nevertheless, for desserts such as ice cream, highly refined peanut oil is generally used, and the likelihood of finding unrefined peanut oil, especially in a vanilla-flavored ice cream, is small. Additionally, in recent years, oral immunotherapy has been actively used in clinical practice to help individuals develop tolerance toward food allergies (Maeda et al., 2022; Tirumalasetty et al., 2023). So, although some risk for unrefined peanut oils exists, it is relatively low.

Taken together, the absence of an explicit indicator for peanut allergens, the generally low risk associated with refined peanut oil, and the probable use of highly refined peanut oil in the fictitious ice cream product, we suggest that the correct answer to question 5 (“Can this product safely be eaten by someone with a peanut allergy?”) should be “yes,” “probably,” or at least “unclear.” Given that 2% of adults in Western nations have a peanut allergy (Lieberman et al., 2021), it is likely they and/or their caregivers would be aware of these considerations and could answer “yes” to this question. Likewise, certain health care professionals and individuals acquainted with these nuances might paradoxically receive a low(er) health literacy rating. This could inaccurately classify individuals, failing to distinguish those well-informed about peanut allergy from those unable to respond accurately due to lower health literacy.

Therefore, we recommend a careful reconsideration of the survey and its application in its current form. Alternatively, a revised and updated label design could include a statement indicating that the product “may contain traces of peanuts” or change the ingredient “peanut oil” to “unrefined peanut oil.”
